# Scaffold Material–Architecture Design Rules Linking Mechanics and Early Osteogenesis in PCL/β-TCP Grid, Honeycomb, and Gyroid Lattices

**DOI:** 10.1021/acsomega.5c10393

**Published:** 2026-04-22

**Authors:** Shweta Thapa, Pete Twigg, Maria Katsikogianni, Dimitra Tsaroucha, Mitali Singhal

**Affiliations:** † Medical & Healthcare Technology, Faculty of Engineering & Digital Technologies, 1905 University of Bradford, Bradford BD7 1DP, England, U.K.; ‡ Faculty of Engineering and Digital Technologies, 1905 University of Bradford, Bradford BD7 1DP, England, U.K.; § School of Chemistry, Faculty of Life Sciences, 1905 University of Bradford, Bradford BD7 1DP, England, U.K.; ∥ Biomedical Science, Faculty of Life Science, 123019 University of Bradford, Bradford BD7 1DP, England, U.K.; ⊥ Institute of Cancer Therapeutics, 123019 University of Bradford, Bradford BD7 1DP, England, U.K.

## Abstract

Clinicians still lack truly patient-specific bone scaffolds that simultaneously match defect mechanics and provide early osteogenic cues because current constructs offer limited control over both lattice architecture and multiscale topography. Here, we compare seven scaffold systems—PCL nanofibrous membranes made by Direct Electrospin Writing (DEW), 3D-printed PCL grid, honeycomb and gyroid lattices, and commercial Ossiform β-TCP grid, honeycomb, and gyroid scaffolds—to derive material–architecture design rules that couple mechanics with early osteogenic response. Scaffolds were fabricated by fused-filament 3D printing or DEW, imaged by SEM/confocal microscopy, mechanically tested in monotonic tension to obtain apparent Young’s modulus and structural stiffness, and cultured with MG63 cells for 7 days to assess cytocompatibility by MTT and morphology. β-TCP scaffolds showed the highest modulus but elastic–brittle failure; printed PCL lattices exhibited tunable viscoelastic behavior, with grid providing the greatest stiffness, honeycomb trading stiffness for energy absorption, and gyroid homogenizing strain. Despite being the most compliant, PCL DEW nanofibers produced the highest early proliferation and most elongated cells, consistent with ECM-mimetic nanotopography, while PCL-gyroid and β-TCP-gyroid supported enhanced spreading and 3D colonization due to continuous curvature and microrough, osteoconductive surfaces. These results indicate that early osteogenic behavior is governed by joint effects of nanoscale topography, curvature-driven strain distribution, and ceramic chemistry rather than stiffness alone. The study is limited to 7-day *in vitro* responses and air-tested mechanics; future work will examine long-term differentiation and mineralization, hydrated/degrading mechanics, and hybrid PCL/β-TCP architectures to translate these design rules toward point-of-care, patient-specific bone repair.

## Graphical abstract

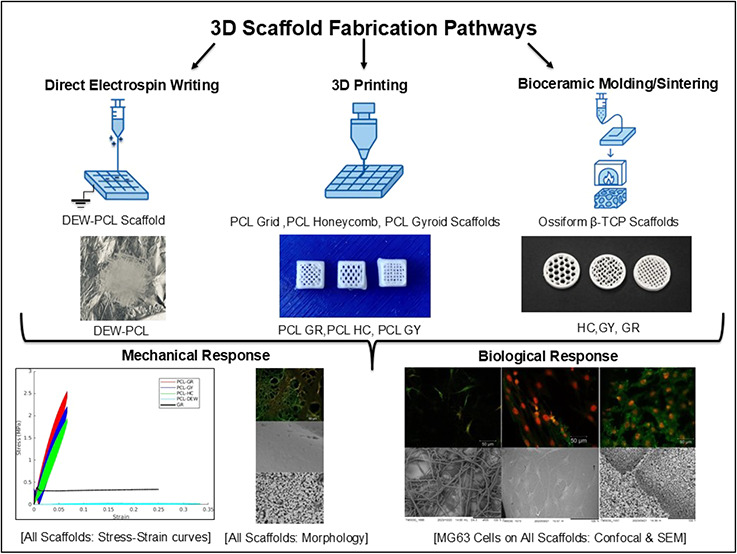

## 1 Introduction

Tissue engineering aims to regenerate, replace, or repair damaged tissues by combining cells with biomaterial scaffolds that provide structural support and instructive biochemical and mechanical cues, as summarized in recent reviews of bone tissue-engineering scaffolds and biomimetic 3D architectures.^1–3^ Scaffold performance depends not only on chemistry but also on architecture—pore size, porosity, and interconnectivity for mass transport; curvature and surface topography for cell guidance and mechanotransduction; and lattice geometry for load sharing and strain distribution—so mechanics and bioactivity jointly recapitulate native microenvironments.^4–7^ Recent work underscores this multiscale view: interconnected porosity tightly regulates cell infiltration and tissue formation, highlighting the need for architecture-aware design in bone repair,^8^ while triply periodic minimal surface (TPMS) designs, such as gyroid, improve mass transport and stress distribution at clinically relevant dimensions.^9^ Dynamically cultured β-TCP scaffolds with large, interconnected pores enhance osteogenic differentiation via improved nutrient and oxygen delivery^10^ and recent reviews emphasize that tailoring PCL architectures—nanofibers, 3D-printed lattices, and hybrid coatings—is essential to couple mechanical competence with cell-material interactions.^3,11^

Despite these advances, there is still no consensus definition of an “ideal” scaffold that standardizes material, shape, surface, and design parameters for a given tissue. Metallic and metal-filled composite systems have been engineered for hard-tissue and cartilage repair, where low-brass-based biomaterials are tuned for pore structure, surface membrane strength, and antitrauma performance under tensile and flexural loads, underscoring how closely mechanical design and clinical function are linked.^24–26^ However, such metallic systems are permanent and can still generate stress shielding or later require removal, whereas degradable polymers and bioceramics could, in principle, provide temporary support and then resorb if their stiffness, architecture, and degradation are appropriately balanced.^2,3^ Polycaprolactone (PCL) is attractive because of its processability and slow degradation, but it is intrinsically bioinert and typically requires architectural or biochemical modification to become osteoinductive.^3,11^ β-Tricalcium phosphate (β-TCP) offers strong osteoconductivity and resorbability, yet brittleness and microstructure-dependent dissolution limit its load-bearing use.^12^ Hybrid PCL/β-TCP constructs seek to combine these advantages,^13^ but systematic, head-to-head comparisons across grid, honeycomb, and gyroid lattices, and across polymeric vs ceramic surface chemistries, remain scarce. Existing comparative and *in vivo* studies often vary materials, print parameters, or assays, making it difficult to extract generalizable design rules, even though gyroid-like architectures have been reported to promote osteogenesis and angiogenesis.^9,14^ In parallel, regulatory frameworks are beginning to support point-of-care manufacture and modular production,^15^ yet there is little guidance on which material–architecture combinations are best suited for patient-specific implants in early osteogenesis.

This study addresses these gaps by systematically comparing seven scaffold systems under a unified experimental protocol: PCL DEW nanofibrous membranes; 3D-printed PCL grid, honeycomb, and gyroid lattices; and commercial Ossiform β-TCP grid, honeycomb, and gyroid scaffolds. We characterize morphology (SEM and confocal imaging), tensile mechanics (apparent Young’s modulus and structural stiffness), and early MG63 cytocompatibility (7-day MTT and morphology) to determine how pattern, material, and surface chemistry together govern stiffness, strain behavior, and early osteogenic response. From these data, we derive practical design guidelines—for example, grid and honeycomb architectures for early fixation stiffness, gyroid for strain homogenization and 3D colonization, and β-TCP chemistry and DEW nanotopography for osteoconductive signaling—providing material–architecture “design rules” for customized, potentially point-of-care, PCL/β-TCP scaffolds in regenerative orthopedics.

## 2 Materials and Methods

### 2.1 Materials

Nanoscale PCL scaffolds were fabricated using polycaprolactone (PCL) pellets (10% w/v, molecular weight 80,000; Sigma-Aldrich), dissolved in methanol and chloroform (2:7) for direct electrospin writing. PCL scaffolds in Grid, Honeycomb, and Gyroid configurations were 3D printed using Facilan PCL 100 (a semicrystalline aliphatic biodegradable polyester) with a molecular weight of 50,000 g/mol manufactured by 3D4Makers, in compliance with European Regulations EC No. 1935/2004. β-TCP scaffolds in Grid, Honeycomb, and Gyroid configurations were sourced from Ossiform, and the MG-63 human osteosarcoma cell line was sourced from Sigma-Aldrich. The L929 cell line (NCTC clone 929: CCL 1; American Type Culture Collection [ATCC], Manassas, VA, USA; ECACC No. 88102702, European Collection of Cell Cultures, Salisbury, Wiltshire SP4 0JG, UK) was also used.

### 2.2 Methods

#### 2.2.1 PCL DEW Scaffolds

The custom-built direct electrospin writing (DEW) setup was used to fabricate fine grid-patterned nanofibrous scaffolds using the optimum operating conditions of 5.75 kV applied voltage, 0.5 mL/h flow rate, and a translation speed of 300 mm/min. The system is interfaced with ReplicatorG software, which allows for manual generation and execution of G-code, controlling the nozzle path and deposition process. A grid structure with an interfiber spacing of 500 μm in both the X and Y axes was encoded into the system for a 5 mm × 5 mm scaffold layout, as shown in Figure S1. A constant layer height of 50 μm and a deposition speed of 300 mm/min were maintained to ensure consistency and control over the fiber architecture.

This method enabled precise layer-by-layer construction, ideal for applications requiring tight control over microarchitecture and porosity, such as neural or dermal tissue engineering.

#### 2.2.2 3D-Printed PCL Scaffolds

PCL scaffolds were designed using SolidWorks and fabricated with polycaprolactone (PCL), a biodegradable polyester widely used in tissue engineering. Scaffolds were modeled with square dimensions of 10 mm × 10 mm × 3 mm. Three infill geometries—Grid, Gyroid, and Honeycomb—were selected for structural variation, as shown in figure S2. Designs were processed using FlashPrint slicing software, which converted the .stl file to gcode, using the following print parameters: layer height of 16 μm, infill density of 50%, nozzle temperature of 170 °C, platform temperature of 45 °C, and print speed of 10 mm/s. Fabrication was carried out using the FlashForge Creator 3 3D printer housed in the Medical and Healthcare Technology Lab at the University of Bradford, using Facilan PCL 100 filament (3D4Makers, Netherlands), which complies with European Regulation EC No. 1935/2004. The material exhibits a glass transition temperature of −60 °C, a density of 1.1 g/cm^3^, and a tensile strength of 45 MPa. Postfabrication analysis revealed challenges in scaffold detachment from the build plate. Scaffolds printed on standard PEI-coated platforms exhibited strong adhesion, necessitating mechanical removal. However, metallic heated build plates facilitated spontaneous release upon cooling, offering a more efficient and less damaging postprocessing workflow.

#### 2.2.3 Ossiform Scaffolds

Commercial scaffolds were sourced from Ossiform, a biotechnology company specializing in bone-mimetic implants. Ossiform utilizes a proprietary 3D printing technique combined with freeze-drying, employing beta-tricalcium phosphate (β-TCP) as the primary material. This results in porous scaffolds with a physiologically relevant architecture suitable for bone regeneration, drug screening, and bacterial or cell interaction studies. Scaffolds were supplied in three distinct architectural designs—Grid, Gyroid, and Honeycomb—to explore how geometry affects cell culture performance and mechanical behavior. The scaffolds have a diameter of 11 mm and an overall cross-sectional area of ∼95 mm^2^.

### 2.3 Morphological Analysis

Morphological characterization of direct electrospun-written nanofiber scaffolds (PCL-DEW), 3D-printed PCL scaffolds in Grid (PCL-GR), Honeycomb (PCL-HC), and Gyroid (PCL-GY) configurations, as well as Ossiform B-TCP scaffolds in Grid (GR), Honeycomb (HC), and Gyroid (GY) configurations, as shown in Figure 1A,B,C, was performed at the Micro and Nanoengineering Technology (MNT) Laboratory using scanning electron microscopy (SEM).

**Figure 1:**
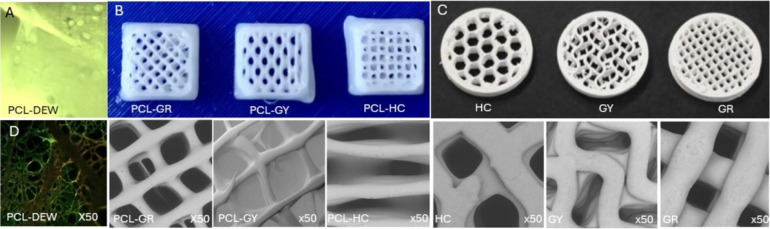
Fabrication overview (macro) of all scaffolds: (A) Direct Electrospun Written PCL scaffold (PCL-DEW). (B) Macro images of 3D-printed PCL scaffolds in grid (PCL-GR), gyroid (PCL-GY), and honeycomb (PCL-HC) configurations for SEM tests. (C) Macro images of 3D-printed β-TCP scaffolds sourced from Ossiform in honeycomb (HC), gyroid (GY), and grid (GR) configurations, respectively. (D) SEM micrographs showing characteristic fiber/pore architectures across scaffold types under 50× magnification.

### 2.4 Mechanical Testing

#### 2.4.1 PCL DEW Scaffolds

A 15 mm × 0.2 mm strip of direct electrospun written (DEW) PCL (area = 3 mm^2^) nanomembrane with a gauge length of 30 mm was clamped between the 2 grips of the Mach-1 mechanical testing system (Model: V500css, Serial No.: MA0561099), equipped with a single-axis 25 kg load cell (SN: 1810278), and controlled via Mach-1 Motion Software Version 4.4.0.17. The system was calibrated prior to testing (calibration factor: 0.994987, offset: −55) to ensure measurement accuracy.

The first phase involved z-stage translating by −10 mm at 0.5 mm/s (loading phase), then holding that displacement for 10 s in the second phase (relaxation), followed by unloading at 0.5 mm/s. One full “ramp” cycle was performed. Raw force (in gf, converted to N) is divided by the cross-sectional area (3 mm^2^) to yield engineering stress (N/mm^2^). Displacement over the original length (30 mm) gives strain. The slope in the initial linear region can be reported as an apparent elastic Young’s modulus (MPa).

#### 2.4.2 3D-Printed PCL Scaffolds

To evaluate the mechanical strength of the 3D-printed scaffolds, rectangular bars (dimensions: 35 mm × 13 mm × 3 mm) were fabricated in each of the three structural configurations (Grid, Honeycomb, Gyroid) using the FlashForge Creator 3 printer and Facilan PCL filament. Mechanical testing was conducted at the Polymer Research Center, University of Bradford, using the Mach-1 Motion System under ramp-release conditions at a velocity of 0.05 mm/s.

#### 2.4.3 Ossiform Scaffolds

To evaluate the mechanical competence of the Ossiform scaffolds, tensile testing was conducted using the Mach-1 Motion Testing System at the Polymer Research Center, University of Bradford. A 25 kg single-axis load cell was employed, and the ramp-release method was used to apply controlled tensile loads at a constant strain rate of 0.05 mm/s. Data acquired from the tests were used for the conversion of load–displacement to engineering stress–strain for the extraction of elastic modulus (slope in the initial linear region), stiffness (machine compliance accounted for if applicable), and identification of the viscoelastic regime (hysteresis/creep relaxation) for the scaffolds.

### 2.5 Cell Culture and Assays

#### 2.5.1 Cytotoxicity/Indirect Cell Culture Testing

The PCL DEW scaffolds, 3D-printed PCL scaffolds, and Ossiform scaffolds were tested for indirect cytotoxicity using the L929 cell line. The cells were cultured in media containing 10% FBS (Sigma-Aldrich, UK), 2.4% l-glutamine (Sigma-Aldrich, UK), and 1% penicillin/streptomycin (Sigma-Aldrich, UK) at 37 °C in 5% CO2. After the cells were grown to confluency, the cell density was adjusted to 1 × 10^5^ cells/mL in a 96-well plate until they formed a half-confluent monolayer, for 24 h prior to exposure to the scaffold-incubated media for a further 24 h of culture. Briefly, the scaffolds were incubated in media for 24 h, and afterward, a 100 μL of the treated media was added to the cells in the 96-well plate for a further 24 h of incubation. Thereafter, the medium was removed again, and 50 μL of MTT (3-(4,5- dimethylthiazol-2-yl)-2,5-diphenyltetrazolium bromide) was added. After 4 h of incubation, the absorbance was recorded at 570 nm using a plate reader.

The cell viability was calculated using the formula Viability % = (100 × OD_570e_)/OD_570_, where OD_570e_ is the mean value of the measured optical density of the 100% extracts of the test sample, and OD_570b_ is the mean value of the measured optical density of the blanks. The lower the Viability % value, the higher the cytotoxic potential of the test item.

#### 2.5.2 Direct Cell Culture Testing

Human osteosarcoma cells (MG-63s) were cultured in Dulbecco’s Modified Eagle’s Medium (DMEM) growth media (Sigma-Aldrich, UK) in T175 flasks containing 10% FBS (Sigma-Aldrich, UK), 2.4% l-glutamine (Sigma-Aldrich, UK), and 1% penicillin/streptomycin (Sigma-Aldrich, UK) at 37 °C in 5% CO_2_. The cells were seeded at a density of 1 × 10^5^ cells/mL on scaffolds and were cultured for days 1, 3, and 7 at 37 °C in 5% CO_2_. At each time point, the media were removed from the well plate and replaced with 500 μL basal media plus 50 μL of MTT (5 mg/mL MTT in PBS). The plates were covered in foil and incubated at 37 °C for 3 h to allow the cells to convert the soluble MTT into insoluble formazan. The media/MTT was then removed, and the formazan was solubilized by a 30 min incubation in acidic isopropanol (0.04 M HCl in 100% isopropanol). All the samples were transferred to a new well plate, and 150 μL of isopropanol solution was added to each sample. The plates were then wrapped in foil and placed on a shaker for 45 min. Then, 150 μL of supernatant from each well containing the polymers and controls was transferred to a 96-well plate. The optical density of the solution at 570 nm (OD570) was measured using a plate reader (ThermoScientific, Multiscan, FC). Cells cultured in dimethyl sulfoxide (DMSO) were considered the positive control.

#### 2.5.3 Scanning Electron Microscopy (SEM)

SEM observations were performed in this study using a FEI Quanta 400 scanning electron microscope for direct visualization of scaffold interactions with cell surfaces. Each cell line was seeded at a density of 1 × 10^4^ cells/ml and cultured for 1, 3, and 7 days at 37 °C in 5% CO_2_. The living cell samples were washed with PBS 3 times after being removed from the cell culture medium. Subsequently, the samples were fixed with 2.5% glutaraldehyde (Sigma-Aldrich, UK) at room temperature for 30 min. Each sample was rinsed with PBS for 15 min (3 times) and then soaked in distilled water for a further 5 min. Finally, the samples were dehydrated in a series of ethanol solutions with 10%, 30%, 50%, 70%, 90%, and 100% (10 min for each concentration). Prior to SEM imaging, all the samples were sputtered with a thin gold film at 20 mA for 1.5 min using an Emitech sputter coater (Quorum Technologies, UK). The scaffolds and controls were securely attached to carbon tabs (Agar Scientific, UK) and placed onto 25 mm SEM specimen stubs (Agar Scientific, UK). The samples were then loaded into the machine for SEM analysis and subsequently analyzed under high vacuum at an accelerating voltage of 15 kV (FEI Quanta 400, Cambridge, UK; XTM version 2.3 software). All images were analyzed using ImageJ software.

#### 2.5.4 Immunofluorescence Microscopy to Image Cell Nucleus and Cytoskeleton—Confocal Scanning Laser Microscopy (CSLM)

To observe the morphology of cells attached to the polymers, the cytoskeleton of cells was stained with two fluorescent dyes: DAPI (4’,6-diamidino-2-phenylindole) and Rhodamine Phalloidin.^16,17^ Each cell line was seeded at a density of 1 × 10^4^ cells/mL and cultured for 1, 3, and 7 days at 37 °C in 5% CO_2_. The samples were then removed from the media and washed with PBS once. Then, the samples were fixed by soaking them in 4% formaldehyde (Sigma-Aldrich, UK) at room temperature for 30 min. Following this, the polymers were washed with 1 mL PBS (3 times) to ensure complete removal of any fixing agent. Afterward, the samples were soaked in 1% Triton-X100 (Fisher Scientific, UK) for 30 min at 25 °C to permeabilize the cells. Three further washes with PBS were performed, and 200 μL of Rhodamine Phalloidin stain was placed onto each sample and incubated for 30 min in a dark room at 25 °C. The Rhodamine Phalloidin (Thermo Fisher Scientific) stain was prepared according to the manufacturer’s instructions. Following this, the samples were washed once with 1 mL of PBS and stained with DAPI (Thermo Fisher Scientific). 50 μL of the DAPI solution was added at room temperature and left for 15 min prior to the final wash in PBS to ensure complete removal of any unbound DAPI and phalloidin conjugates. The samples were wrapped in foil, as they are light-sensitive prior to imaging. A Zen microscope was used for direct visualization of cell-seeded samples. All images were analyzed using the ImageJ software for DEW PCL Scaffolds, 3D-Printed PCL Scaffolds, and Ossiform P3D Scaffolds, respectively. Aspect ratio calculations from the SEM images were also carried out using ImageJ for the cells from day 1 to day 7 to better understand the cell behavior on the scaffold.

Aspect Ratio = Cell Length (major axis)/Cell Width (minor axis)

A perfectly round cell has an aspect ratio close to 1. As cells elongate, this value increases.

## 3 Results and Discussion

### 3.1 Morphological Analysis

#### 3.1.1 PCL DEW Scaffolds

At a magnification of 50×, the scaffolds exhibited a highly organized and linear fiber alignment, closely emulating the architecture of the native extracellular matrix (ECM), as seen in Figure 1D. This ordered arrangement is indicative of the scaffold’s potential to support contact guidance and direct cellular orientation during tissue regeneration. Higher magnification (50×) SEM imaging in Figure 1D revealed a densely interconnected fibrous network with well-defined porosity. Pore diameters within the scaffold ranged from 10 to 40 μm, suggesting an architecture favorable for nutrient diffusion, metabolic waste exchange, and cell infiltration—key requirements for tissue engineering applications. The nanofibers themselves exhibited a uniform diameter distribution, with measurements taken from ten randomly selected fibers using ImageJ software. The average fiber diameter was determined to be 1.8 μm, with a standard deviation of 0.48 μm, as seen in Figure 2A. The fibers frequently formed bifurcating, branch-like patterns, contributing to the overall structural complexity and mechanical interconnectivity of the scaffold. The observed morphology highlights the scaffold’s biomimetic nature and its suitability for applications requiring anisotropic structural cues, such as nerve, tendon, or muscle tissue engineering.

**Figure 2:**
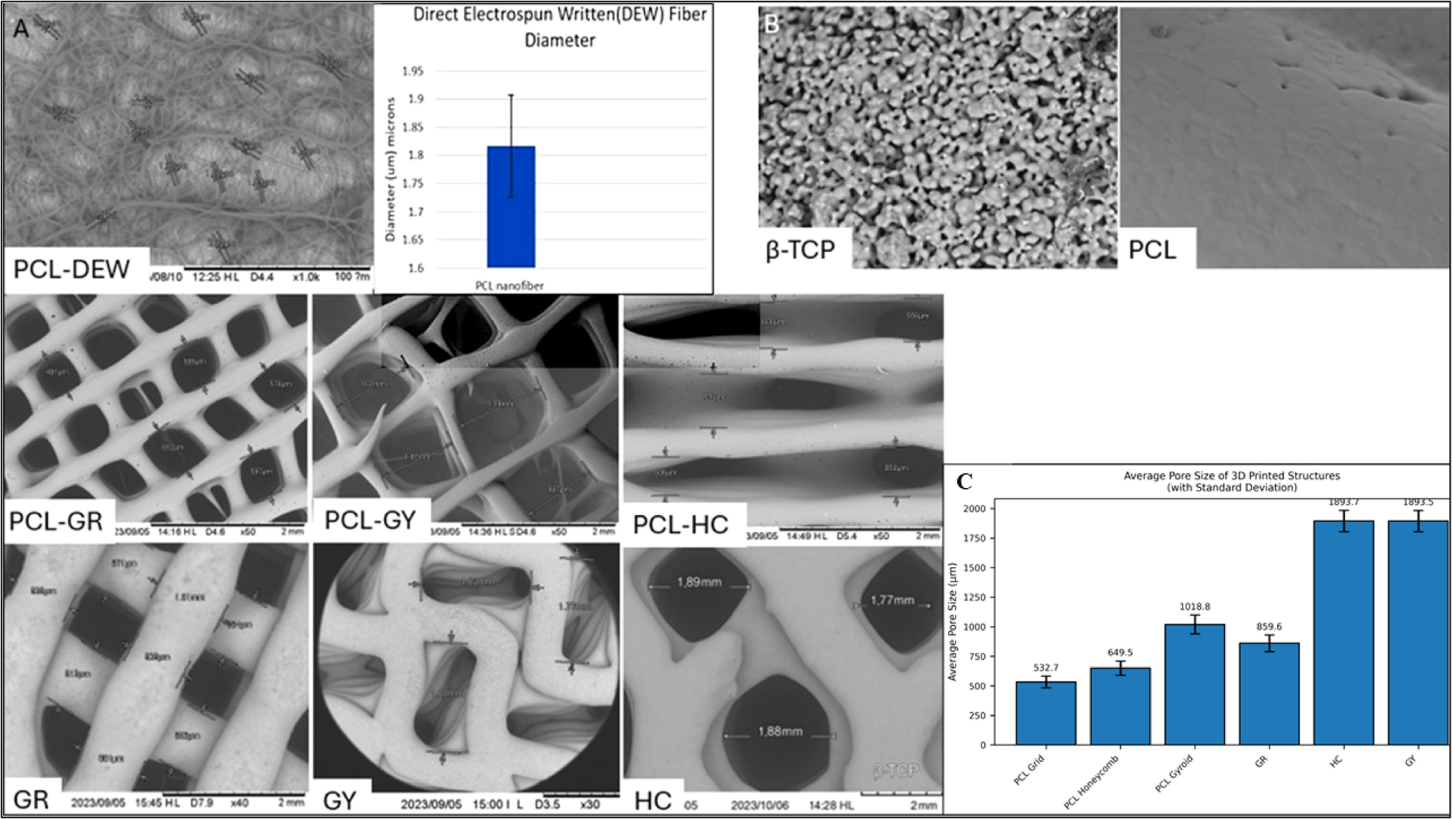
High-magnification SEM and quantitative pore-size analysis across all scaffolds: (A) SEM micrograph showing direct electrospun written PCL-DEW nanofibers (left) and their measured average fiber diameters (Right). (B) Surface roughness/microtopography (β-TCP vs PCL close-ups) 2000× magnification. (C) Pore size quantification for all 3D-printed scaffolds: PCL-grid­(PCL-GR), honeycomb (PCL-HC), and gyroid (PCL-GY) as well as Ossiform-grid (GR), gyroid (GY), and honeycomb (HC).

#### 3.1.2 3D-Printed PCL Scaffolds

SEM analysis revealed that all scaffold types exhibited a porous, interconnected topology, essential for effective nutrient transport and cellular infiltration. However, visual inspection of the printed PCL scaffolds (Figure 1B) revealed noticeable variations in pore shape and size across the different lattice architectures. The honeycomb PCL-HC design exhibited the greatest degree of nonuniformity, characterized by irregular pore boundaries, local strut thickening, and deformation at node intersections. In contrast, the grid PCL-GR and gyroid PCL-GY structures displayed comparatively more consistent pore geometry, as seen in Figure 1B. These irregularities in the honeycomb scaffolds appear to result from a combination of printing-related factors and geometric design sensitivity. Specifically, fluctuations in extrusion flow and localized cooling effects likely contributed to strut over- or underdeposition, while the angled trajectories and tight node radii inherent to the honeycomb pattern amplified these deviations. Together, these effects produced variable pore dimensions across the construct. Improving thermal and pressure control during printing, reducing speed, and optimizing node thickness can significantly enhance pore uniformity. Quantitative analysis of pore dimensions across the different patterns, as seen in Figure 1C indicated: Grid pattern: Pore sizes ranging between 0.4 and 0.6 mm; Honeycomb pattern: Pores spanning 0.5–1.0 mm; Gyroid pattern: Larger pores measuring approximately 0.9–1.2 mm. The pores were measured using ImageJ software to average out the pore size for PCL grid ∼523 μm, PCL honeycomb ∼641 μm, and PCL gyroid ∼1082 μm, with standard deviations of ±59.1 μm, ±86.1 μm, and ±79.2 μm, respectively.

#### 3.1.3 Ossiform P3D Scaffolds

The macro 3D-printed Ossiform scaffolds are shown in Figure 1C. Scanning electron microscopy (SEM) imaging at a magnification of 2000× revealed a granular surface microstructure, as shown in Figure 2B. These surface granules, distributed uniformly across the scaffold, significantly contribute to surface roughness and microporosity, both of which are favorable for osteoconduction and cellular attachment. The presence of such microtextured surfaces enhances the scaffold’s potential to support cell adhesion and proliferation by mimicking the hierarchical topography of natural bone. The PCL surface, on the other hand, is smooth, not offering as many focal points for gripping. Additionally, the porosity observed at both the micro- and macrostructural levels is essential for facilitating nutrient transport, vascularization, and bone ingrowth.

Collectively, these observations reveal a trade-off between architectural precision and bioactive topography. PCL DEW provides highly ordered, ECM-like fibrous cues but low intrinsic stiffness; 3D-printed PCL lattices offer tunable macroporosity yet relatively smooth struts and, for honeycomb, print-driven pore variability; Ossiform β-TCP combines regular macropores with microroughness but is limited by ceramic brittleness.

### 3.2 Mechanical Characterization

#### 3.2.1 PCL DEW Scaffolds

Preliminary mechanical analysis revealed an elastic Young’s modulus in the range of 0–0.5 MPa, indicative of a compliant and flexible material. At several points, it is observed that stress drops abruptly at nearly constant strain, indicating the viscoelastic nature of relaxation. There’s is also evidence of softening at higher strain, showcased by a gradual decline in the Young’s modulus, as seen in figure S3. The presence of elastic and viscoelastic features allows the nanomembrane to not only support loads but also dissipate stress, building a dynamic mechanical environment suitable for guiding cell behavior and tissue formation, especially for soft tissues like skin, cardiac, or neural extracellular matrix.

Due to the delicate and thin nature of the electrospun written scaffolds, the use of a lower-capacity load cell is recommended in future studies to enhance measurement sensitivity and reduce the risk of mechanical failure during testing. Although such properties are beneficial for certain soft tissue applications, fabricating thicker scaffolds may be necessary to more closely replicate the mechanical profile of native connective tissues, particularly in load-bearing environments. Recent overviews of collagen- and gelatin-based electrospun fibers report moduli in the low-MPa range, strongly dependent on fiber diameter, mat thickness, and cross-linking, consistent with the high deformability of very thin nanofibrous membranes.^18,19^ Likewise, studies on ultrathin polymer films show that thickness in the tens-to-hundreds-of-nanometers regime significantly alters the effective Young’s modulus compared with thicker films, with single- or tens-of-MPa ranges commonly reported for soft, free-standing films.^20,21^ Finally, viscoelastic stress relaxation and creep, similar to that observed here, are widely documented in hydrogel and hydrogel–fiber systems, where tunable relaxation rates govern the balance between elastic recovery and time-dependent deformation.^22,23^

#### 3.2.2 3D-Printed PCL Scaffolds

The primary objective of the test was to determine the modulus of elasticity and stiffness of each scaffold type, as these parameters are essential indicators of load-bearing capacity and elastic behavior. The slope of the linear region of the viscoelastic load–displacement curves was used to calculate stiffness, while the initial slope of the stress–strain curves provided values for the elastic modulus. Results from mechanical testing demonstrated a significant influence of the infill pattern on the scaffold’s mechanical properties:

PCL Grid-patterned scaffold exhibited the highest modulus of elasticity (∼50 MPa), reflecting superior stiffness and load distribution due to its organized and rectilinear structure. Linear elastic response is observed up to ∼2% strain with a proportional stress–strain relationship; however, after that, nonlinear viscoelastic/plastic behavior is visible, typical of viscoelastic polymers, exhibiting hysteresis on unloading, as seen in figure S4. PCL Gyroid-patterned scaffold showed intermediate stiffness, with a more complex, continuous architecture supporting both structural resilience and flexibility. Its continuous, 3D-woven topology provides a more uniform load distribution, leading to a smoother transition into viscoelastic flow. It delays the onset of nonlinear (viscoelastic) behavior to ∼3% strain, vs ∼2–2.5% for the others. Its continuous curvature reduces local stress peaks, so polymer chains remain in the linear regime longer under a ramp load. PCL Honeycomb pattern recorded the lowest modulus (∼41 MPa), attributed to its larger pores and more material-efficient hexagonal configuration. Postyield behavior shows stress relaxation and nonlinear stiffening, likely due to filament bending and interstrand interactions in the honeycomb topology. The honeycomb pattern yields a lower modulus compared to a solid grid of the same density (grid was ∼50 MPa), reflecting its more compliant structure under load. Viscoelastic effects emerge beyond the elastic limit, as seen in the curvature of the stress–strain plot (refer to Figure 3A for a comparison between different scaffold types).

**Figure 3:**
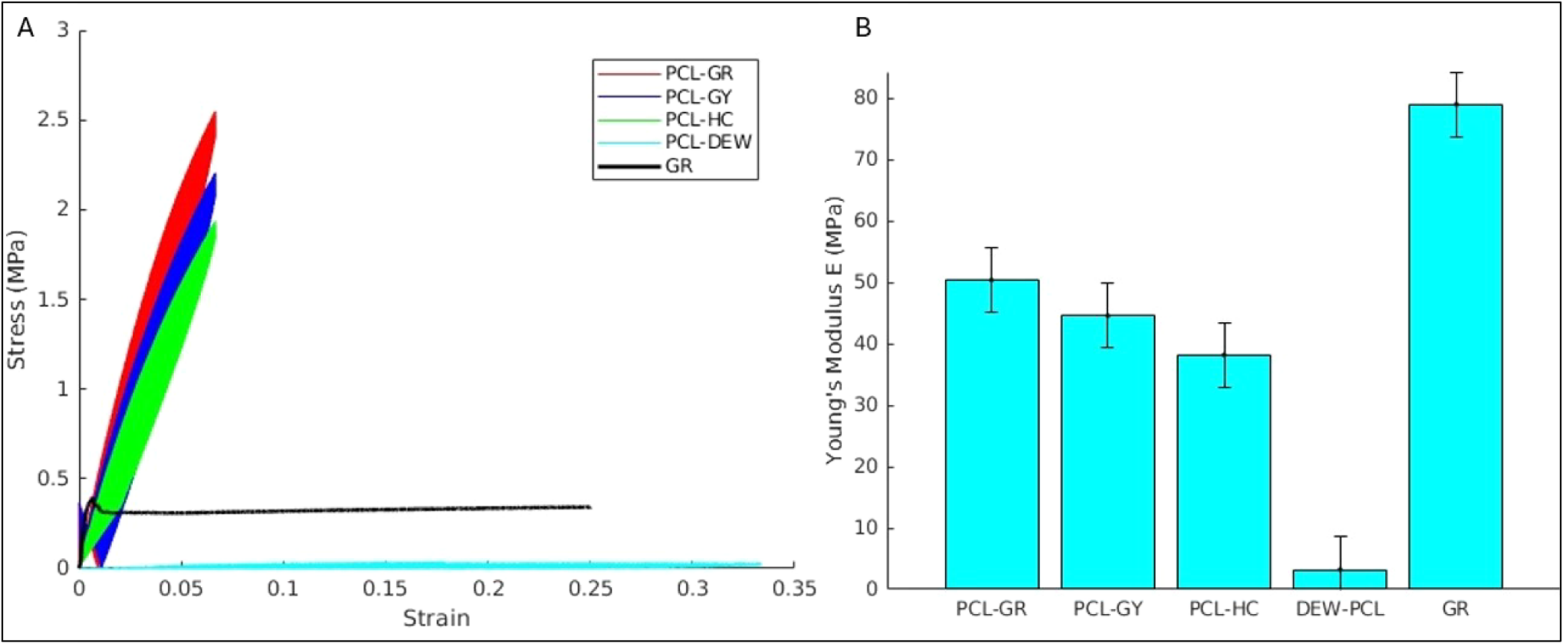
Mechanical behavior of PCL-DEW, 3D-printed PCL lattices (PCL-GR, PCL-GY, PCL-HC), and β-TCP scaffold (GR): (A) Representative stress–strain curves. (B) Elastic modulus and structural stiffness comparisons (mean ± SD).

Sharp angles in Grid and Honeycomb induce stress raisers, promoting earlier chain sliding (viscoelasticity). The Gyroid’s absence of sharp corners smooths stress flow, enhancing elastic range at the expense of overall stiffness. These mechanical differences highlight the impact of internal architecture on scaffold performance. While higher infill density generally contributes to increased stiffness, geometry itself is equally pivotal in modulating mechanical response. The grid configuration, for instance, provides enhanced resistance to deformation due to its uniform strut alignment, whereas the honeycomb design prioritizes material efficiency and isotropic behavior. Under tensile loading, grid-pattern specimens exhibit the highest ultimate tensile strength compared with triangular and honeycomb infills (56–72 MPa vs ∼27 MPa for quarter-cubic).^27^ Honeycomb typically trades off stiffness for improved energy absorption and ductility, known to fail in a ductile manner with its filaments necking down, whereas grid fails more brittlely.^28,29^ In fact, for fiber-reinforced polymers, honeycomb at high infill densities delivers a very good tensile modulus (∼4.8 GPa) but still falls below grid/triangular in ultimate strength.^27–29^

This study reaffirms the tunability of mechanical properties in 3D-printed PCL scaffolds through the strategic selection of infill patterns, elastic modulus, and stiffness, as detailed in Figure 3 for all scaffolds. By tailoring architectural parameters, it is possible to design scaffolds that balance mechanical integrity with biological functionality, thus advancing the development of application-specific tissue engineering platforms.

Ossiform β-TCP scaffolds: The elastic modulus, Figure 3B, indicates that the bioceramic scaffolds demonstrated the highest stiffness among all scaffolds in the linear range but exhibited brittle failure characteristics. In the linear elastic region, the scaffolds withstood tensile stresses in the range of 70–80 MPa before catastrophic failure (for more details, refer to S5). There was no significant plastic deformation prior to fracture, reflecting the inherent brittleness of β-TCP-based scaffolds. This brittle behavior is attributed to several factors, primarily the intrinsically low fracture toughness of the β-TCP ceramic material. While the high modulus and compressive resistance of bioceramics make them suitable for nonload-bearing or low-load-bearing bone repair, their limited energy absorption capacity and susceptibility to brittle fracture restrict their application in dynamic or high-stress environments without reinforcement. These results underscore the trade-off between mechanical strength and fracture resistance in porous bioceramic scaffolds and highlight the need for design optimization or composite strategies (e.g., polymer–ceramic hybrids) to enhance toughness while retaining bioactivity.

Mechanical testing was performed in air; however, PCL softens only modestly when hydrated, and β-TCP ceramics remain mechanically stable. Therefore, while absolute moduli may be slightly lower under wet conditions, the stress–strain profiles and stiffness hierarchy shown in Figure 3A,B remain preserved and are representative of the relative mechanical environment experienced by cells during culture.

In summary, each scaffold family occupies a distinct mechanical niche rather than approaching an “ideal” profile. PCL DEW nanomembranes are highly compliant and viscoelastic, suitable for soft-tissue or interface applications but unable to provide structural fixation alone; 3D-printed PCL lattices tune stiffness via architecture (grid → highest modulus but sharper stress raisers, honeycomb → lower stiffness but better energy absorption, gyroid → extended elastic range with smoother stress distribution but reduced peak stiffness); and Ossiform β-TCP offers the greatest stiffness but brittle, low-strain failure, limiting its role to osteoconductive support rather than stand-alone load-bearing. Collectively, these findings argue for hybrid designs that combine a stiff architectural core with nano/micro-topographical cues, and they indicate that the stiffness hierarchy observed in air—largely preserved in culture—will meaningfully shape the osteogenic responses reported in the following sections.

### 3.3 Biological Characterization: Cell Morphology, Viability, and Regeneration

All scaffold types (PCL-DEW, 3D-printed PCL: PCL-GR, PCL-GY, PCL-HC; and Ossiform β-TCP: GR, GY, HC), after undergoing indirect cytotoxicity tests as described in the methods above, remained well above the commonly used 70% viability threshold for noncytotoxic materials. Results demonstrate that the scaffolds are noncytotoxic under indirect exposure conditions and suitable for subsequent direct cell culture studies (Figure S6). PCL-DEW nanofibers: The SEM image of direct electrospun written (DEW) scaffolds after 1 and 7 days of direct cell culture with Mg63 human osteosarcoma cells showcases progressive changes in cell morphology, distribution, and elongation. Figure 4A Day 1, 600× magnification highlights rounded Mg63 cells with minimal spreading and limited filopodia extension, indicating early adhesion phases. The cell bodies are sparsely distributed and loosely interacting with the nanofibrous direct electrospun written nano scaffold grid. An average aspect ratio of 1.4–1.7 is calculated from the 1000× Day 1 magnified image (Figure S7). Day 3 cell growth (Mg63 cells) and interaction on the scaffolds are visible with confocal imaging, cell morphology, and locations (Figure 4 H1).

**Figure 4:**
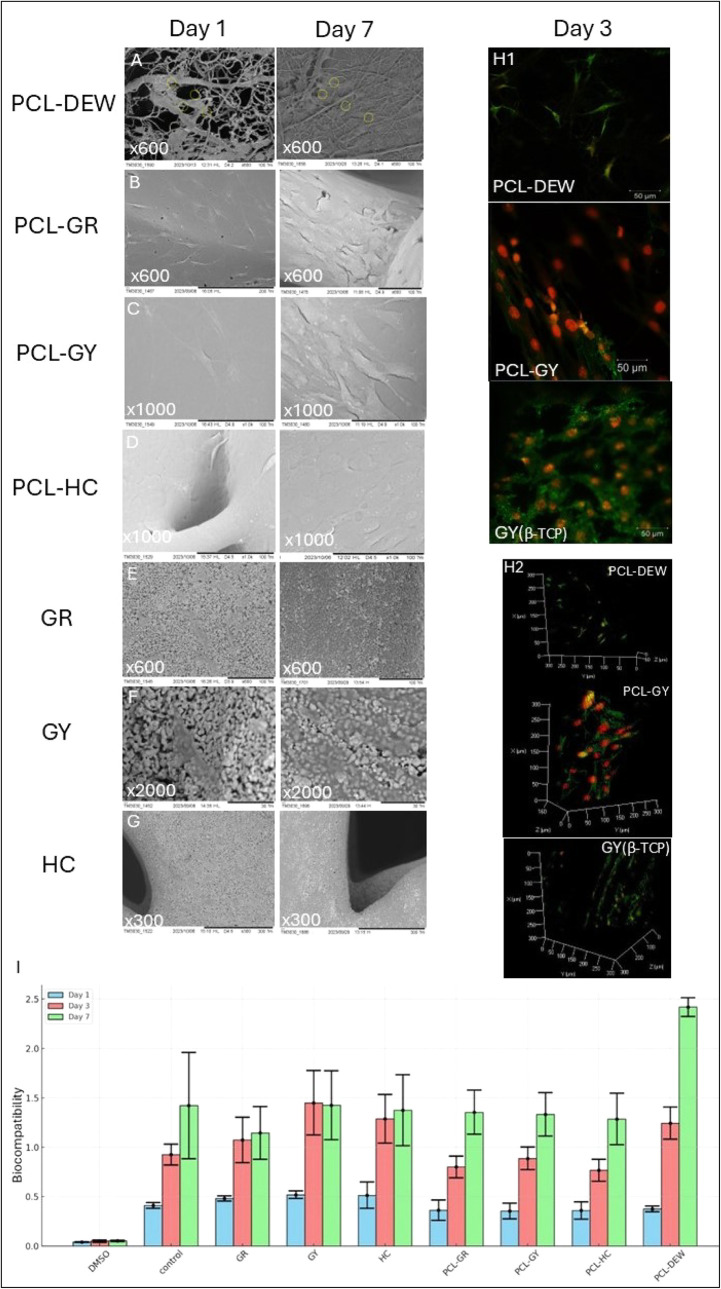
Cell morphology and spreading on scaffold surfaces: (A–B–C–D–E–F–G) SEM micrographs showing attachment and elongation on PCL-DEW (A), 3D-printed PCL lattices (B, C, D), and granular attachment on β-TCP scaffolds (E, F, G). H1 Confocal images (Day 3) display nuclei (DAPI) and actin cytoskeleton (phalloidin) on scaffold surfaces. H2 Orthogonal views showing 3D colonization for PCL-DEW, 3D-printed PCL-gyroid, and β-TCP gyroid, respectively. (I) MTT assay (MG63, Days 1, 3, and 7) showing percentage cell viability on each scaffold.

Day 7, 600× magnification images exhibit confluent-like growth, bridging across multiple fiber strands, confirming robust scaffold integration and proliferation. This suggests stronger cytoskeletal organization and scaffold-guided contact guidance. The Day 7 average Aspect Ratio = ∼3.5–5.0 when measuring the Day 7 1000× magnified image (Figure S7) indicating highly elongated cells aligned with nanofiber orientation. Figure 4I shows the cell viability assessment via the MTT assay for MG63 human osteosarcoma cells on the nanoprinted PCL-DEW pattern against all other scaffolds. It provides a metabolic activity-based measure of cell viability. PCL-DEW shows marginally reduced viability compared to Control Day 1, possibly due to initial adaptation and delayed adhesion dynamics on the PCL-DEW structure, as reinforced by the SEM images in Figure 4A, where cells appear less spread and globular. The Day 7 DEW-PCL scaffold, however, surpasses both Control and DMSO in viability, suggesting enhanced proliferation following the initial lag (Figure 4I). This is further reinforced by the morphological observations seen in Figure 4A Day 7 600× magnification, where extensive cell spreading and scaffold bridging are apparent, reflecting increased cell proliferation and metabolic activity. Additionally, the immunofluorescent and 3D image of the PCL-DEW scaffold on Day 3 (Figure 4H2) reveals several useful details on cell morphology, spreading, and the behavior of the cytoskeleton over the 3-day period in the 3D scaffold. The green fluorescence indicates the F-actin staining via phalloidin (refer to Methods – Cell to Culture and Assays) representing the elongating cytoskeleton of the osteosarcoma cell. The actin fibers appear well-organized and elongated along scaffold features, suggesting that the PCL-DEW scaffold induces contact guidance, forcing cells to align and elongate in response to the scaffold’s topography. By Day 7, the high density of green fluorescence across the image implies that cells are spreading across and along the nanofibers, covering scaffold surfaces, as seen in the orthogonal views and z-stacks (Figure S10), confirmed by the increased viability observed in the MTT assay (Figure 4I). The blue spots due to the DAPI staining represent the nuclei, which appear to be elongated or elliptical, consistent with cytoskeletal elongation and tension transfer from the scaffold to the nucleus, as well as the known integrin–cytoskeleton–YAP/TAZ mechanotransduction cascades.^30^

The orientation of nuclei aligns with actin filaments, which is typical for cells sensing and responding to scaffold architecture—in this case, the aligned nanofibers—another indicator of mechanotransduction effects from the PCL-DEW (Figure 4H1). The pronounced cell elongation and alignment observed on the PCL-DEW scaffold in our SEM (Days 1 and 7) and confocal imaging (Days 3 and 7) are consistent with classical contact-guidance behavior on anisotropic micro–nano topographies. Such oriented features promote integrin clustering and focal-adhesion maturation, which organize actin stress fibers acting along the scaffold axis and increase cytoskeletal tension.^31^ Importantly, aligned fibers have been shown to enhance nuclear localization of YAP/TAZ, linking topographical guidance to transcriptional programs that regulate proliferation and cytoskeletal organization.^32,33^ These mechanisms collectively support our observations of increased spreading, directional growth, and higher viability on PCL-DEW in the MTT assay, suggesting that the scaffold’s aligned architecture not only directs morphology but also enhances mechanosensitive signaling that promotes cell survival and organization.

The combined morphological (cell elongation, scaffold integration) and biochemical (MTT assay) data indicate that the directly electrospun written PCL-DEW scaffold effectively promotes cell adhesion, proliferation, and alignment over time, with promising potential for long-term cell viability and spreading. PCL-DEW’s high proliferation is driven primarily by its nanoscale topography rather than its lower stiffness, reflecting ECM-like fiber dimensions that strongly promote early adhesion and cytoskeletal organization.

#### 3.3.1 PCL 3D-Printed Scaffolds

3D-printed scaffolds (10 × 10 mm^2^, 3 mm thick) in grid, honeycomb, and gyroid patterns, respectively, were fabricated using Polycaprolactone (PCL) filament and the melt extrusion process (FDM) in a Flashforge Creator 3 3D printer at WG37, University of Bradford. After successful indirect cytotoxicity results, the scaffolds were cultured for direct testing using MG63 human osteosarcoma cells (refer to Methods – Cell Culture and Assays) for 1, 3, and 7 days, respectively, to better understand the proliferation, viability, and cell growth dynamics over the surface of the scaffold.

The initial adhesion phase is evident on day 1 for all PCL 3D-printed scaffolds. Referring to Figure 4B,C,D, the surface appears smooth with clear, defined PCL fiber structures. Few cells are present, appearing round and not fully spread (Figure 4H1 PCL-GY); no extended filopodia are seen, and cell distribution seems sparse. The PCL-GR scaffold highlights elongated cells with longer, directed filopodia, suggesting quicker engagement with the grid’s linear features. Day 1 images for PCL-GY showcase that some cells have already penetrated scaffold channels, hinting at better surface interaction. There are minimal signs of degradation or surface pitting. The PCL-HC scaffold shows sparsely attached cells with rounded to mildly spread morphologies, extending only a few short filopodia toward scaffold walls. Cells are isolated, and few bridge the adjacent honeycomb walls. A high aspect ratio (∼1.26) is observed across all day 1 SEM images of PCL-GY, indicating consistent fabrication and geometry.

Day 7 SEM images highlight spreading, elongation, and dramatically enhanced cell coverage for the PCL-GR and PCL-GY scaffolds, with numerous bridging connections across fibers. Cells are flattened and spindle-shaped, following the scaffold geometry’s linear architecture. The PCL-GR side views show deeper infiltration and possible deposition of extracellular matrix-like material. There are noticeable degradation features, such as slight fiber thinning and possible merging or cracking on the scaffold surface. Fibers appear less distinct, suggesting ongoing surface erosion or polymer breakdown. A highly spread morphology with interconnected cells forming a network, along with extensive filopodia extensions and possible early tissue-like organization, is observed. ECM presence is more prominent, possibly contributing to fiber masking in the PCL-GR and PCL-GY scaffolds.

The PCL-HC scaffold Day 7 image does not show the strong uniaxial alignment pattern evident in PCL Grid samples. Cell morphology seems more spread and random, consistent with the honeycomb design that lacks straight guiding cues. For the PCL-GY scaffold on Day 7, a notable increase in aspect ratio (1.66) is likely due to cell proliferation filling voids or structural deformation under biological load. It is evident that cells favor bridge formations, smooth curves, and pore intersections, enhancing infiltration depth compared to PCL Grid and Honeycomb. Cell viability trends from the MTT assays in Figure 4I supported these morphological findings. The Gyroid scaffold showed the highest cell proliferation across all time points, with significant differences compared to the Grid and Honeycomb scaffolds by Day 3 and Day 7. SEM micrographs revealed that cells on Gyroid scaffolds exhibited superior elongation, forming stretched and well-spread morphologies that adhered effectively along the scaffold’s undulating surfaces, suggesting enhanced cytoskeletal organization and directional spreading within the 3D environment. The Day 3 colonization of MG63 cells is visible in the z-stack reconstructed 3D confocal image (Figure 4H2, PCL-GY). The complex curvature of the gyroid architecture likely provides a microenvironment that facilitates multidirectional cell attachment and migration, features known to promote osteogenic differentiation and tissue integration.^33^

In contrast, PCL-GR scaffolds promoted linear cell alignment along the struts, resulting in an increase in aspect ratio from 1.4 to 2.1 by Day 7. However, the flat, planar nature of this geometry limited the extent of 3D colonization and lateral spreading, which was reflected in comparatively lower cell viability values at Day 3 and Day 7 in the MTT assay. The Honeycomb (PCL-HC) design demonstrated moderate performance, with cells exhibiting aspect ratios rising from 1.3 to 1.8, indicating partial alignment along the hexagonal channels but with limited depth of colonization relative to the gyroid scaffold. These results underscore the advantages of continuous and smoothly curved architectures in enhancing cellular responses, further resonating with previous reports suggesting that biomimetic curvature and pore interconnectivity promote nutrient diffusion, cell migration, and tissue integration in 3D-printed bone scaffolds.^34^ Cell spreading on PCL-GY is driven primarily by its continuous curvature rather than stiffness, enabling curvature-guided cytoskeletal organization characteristic of TPMS surfaces.

#### 3.3.2 Ossiform P3D Scaffolds

Ossiform P3D scaffolds in configurations of Grid (GR), Gyroid (GY), and Honeycomb (HC) are fabricated using 3D printing (FFF) from a paste of β-TCP, which is later freeze-dried. These commercially available scaffolds were directly tested for cell culture with MG63 human osteosarcoma cells for 1, 3, and 7 days, respectively, and the results look promising with respect to osteogenic differentiation. SEM analysis Figure 4E-F-G revealed clear distinctions in cell morphology and attachment across scaffold designs. On Day 1, cells on the GR (Grid) and HC (Honeycomb) scaffolds appeared less spread, predominantly round, with a lower aspect ratio (∼1.3–1.4), indicating initial cell adhesion without pronounced spreading. In contrast, GY (Gyroid) scaffolds demonstrated superior early attachment, with cells displaying a spindle-like, elongated morphology (aspect ratio ∼2.0), suggesting enhanced cytoskeletal tension and spreading. By Day 3, all scaffolds supported more extensive cell spreading . GY scaffolds consistently displayed the highest aspect ratios (∼2.5), indicating continued elongation and contact guidance along the interconnected curved surface features (refer to 4H1 GY (β-TCP), confocal image for cell image and distribution). In contrast, GR and HC scaffolds promoted isotropic spreading (aspect ratios ∼1.5–1.7), likely due to their planar and repeating geometrical units offering limited guidance cues. The trend seems to persist even on Day 7, whereby GY scaffolds facilitated the highest cell elongation and directional alignment (aspect ratio ∼3.1 ± 0.3), while HC and GR exhibited statistically lower aspect ratios (∼1.6–1.9).

SEM images also highlight that cellular bridging was most evident on GY scaffolds, where interconnected porous structures allowed cells to span across adjacent filaments, promoting the formation of a confluent cell layer over time, compared to HC and GR scaffolds.^35^ Further supporting this finding, the results highlight the effect of surface curvature on cell area and motility

The MTT assay data, Figure 4I, demonstrated temporal trends consistent with SEM observations:

Day 1: Cell viability was low but comparable across all scaffolds. GY exhibited slightly higher metabolic activity, suggesting enhanced early adhesion and spreading.

Day 3: A significant increase in cell viability on all Ossiform scaffolds was observed. GY showed the highest OD_570_ values (∼1.5), followed by HC (∼1.2) and GR (∼1.0). This aligns with SEM evidence of greater cell coverage and spreading on GY at Day 3.

Day 7: The metabolic activity plateaued or declined slightly for GY but remained higher than GR and HC. GY maintained the highest viability, indicative of sustained proliferation and metabolic activity. Notably, GR showed the lowest OD_570_, supporting the SEM findings of limited cell spreading and coverage. These observations suggest that GY scaffolds offer superior support for osteosarcoma proliferation over time, potentially due to their high surface area and continuous curvature enabling favorable mechanotransduction signaling.^36^ Additionally, the larger SD values (error bars) for some conditions reflect biological variability in MG63 colonization of 3D scaffolds (especially β-TCP gyroid and PCL-DEW), rather than measurement errors.

The cellular morphology observed on the Ossiform Gyroid β-TCP scaffold suggests activation of curvature-dependent mechanotransduction pathways. SEM and confocal imaging show that cells readily anchor to the microrough β-TCP surface, promoting integrin clustering and focal adhesion formation. As cells spread along the curved gyroid surfaces, they develop actin stress fibers consistent with increased cytoskeletal tension and FAK–RhoA/ROCK signaling. Although the gyroid structure is not linearly aligned, its continuous curvature provides topographical contact guidance that drives partial elongation and directional organization. These tension-dependent behaviors are well known to favor YAP/TAZ activation, and the cell spreading, bridging of pore walls, and multilayer distribution seen in the Day 3 and Day 7 z-stack images are consistent with such mechanosensitive responses. Collectively, these observations explain the enhanced metabolic activity detected in the MTT assay and strongly suggest that the gyroid β-TCP architecture promotes cell proliferation through integrin-mediated mechanosensing rather than fiber alignment-based guidance.

The immunofluorescent 3D reconstructed z-stack image of Ossiform β-TCP grid scaffold (day 3), Figure 4H2 GY­(β-TCP), highlights robust cell attachment and spreading not only on the flat strut surfaces but also seems to extend into the pore regions, which is encouraging for 3D colonization. Distinct spots—the nuclei (blue, DAPI)—are visible along the scaffold struts, suggesting successful adhesion with some clustering along the edges. Cytoskeletal staining, actin filaments (green, phalloidin), look well-spread rather than just rounded or weakly attached cells. Overall, good osteoconductivity and viability with MG63 cells is evident. In the Day 7 GY­(β-TCP) scaffold, the isolated spherical DAPI signal (figure S9) corresponds to a nucleus positioned within a deep gyroid pore. z-Stack and orthogonal reconstruction confirm that the nucleus is present across multiple optical planes, consistent with a true cellular structure rather than an imaging artifact. The gyroid’s curved pore geometry frequently positions cells in recessed regions where only the nucleus is visible in a given z-section, explaining the apparent localization pattern (figure S11).

Comparative Analysis: The direct cell culture results for all the scaffolds with MG63 osteosarcoma cells have been extremely helpful in shedding light on the different types of scaffold materials being used in the project and understanding their efficacy. The cell viability graph (Figure 4I) highlights clear differences between the Ossiform scaffolds (GR, GY, HC) and the corresponding in-house 3D-printed PCL structures (PCL-GR, PCL-GY, PCL-HC), together with the Direct Electrospun Written PCL-DEW scaffolds. A comparative analysis of MG63 behavior over 7 days showed scaffold-dependent variations in adhesion, morphology, and proliferation. Table 1 below summarizes the key observations for all scaffold types.

**Table 1:** Overview of Scaffold Material, Architecture, Apparent Tensile Modulus, and Qualitative Early Osteogenic Response (MG63, Day 7)

Scaffold code	Base material	Architecture/scale	Apparent Young’s modulus *E* (MPa, mean ± SD) *	Failure mode (tension)	Early MG63 response (Day 7) – qualitative summary
PCL-GR	PCL	3D-printed grid; rectilinear struts (macro-porous)	∼50 ± 4	Ductile; linear elastic region followed by viscoelastic yielding and gradual strut fracture	Moderate proliferation: cells align along struts with limited bridging between pores; mostly spindle-shaped but constrained to macro-tracks.
PCL-GY	PCL	3D-printed gyroid (TPMS); continuous curvature	∼44 ± 4	Ductile; distributed strain and progressive damage, good energy absorption	Sustained proliferation: cells show elongated morphology and wrap around curved struts; improved spreading and 3D colonization vs grid/honeycomb.
PCL-HC	PCL	3D-printed honeycomb; hexagonal cells	∼38 ± 4	Ductile; efficient load sharing along cell walls	Moderate proliferation: cells span across hexagonal windows but with less guidance than gyroid; intermediate spreading.
PCL-DEW	PCL	Direct Electrospin written nanofibrous membrane; aligned fibers (submicron)	∼3 ± 5	Highly compliant; large strain to failure, fiber pull-out and necking	Highest early proliferation: cells highly elongated and aligned along fibers with dense coverage, consistent with ECM-like nanotopography.
GR (β-TCP)	β-TCP Bioceramic	Commercial Ossiform grid-like porous ceramic	∼78 ± 6	Elastic–brittle; linear response and low strain to catastrophic fracture	High proliferation and robust adhesion; cells adopt spreading, membrane-like morphology over microrough ceramic, reflecting strong osteoconductivity.
GY (β-TCP)	β-TCP Bioceramic	Commercial Ossiform gyroid-like porous ceramic	(similar to GR; slightly lower due to geometry, e.g., ∼70 ± 6)	Elastic–brittle; failure initiated at curved strut junctions	High proliferation: cells infiltrate pores and show 3D distribution, combining ceramic bioactivity with curvature-guided spreading.
HC (β-TCP)	β-TCP Bioceramic	Commercial Ossiform honeycomb-like porous ceramic	(similar order to GR, e.g., ∼75 ± 6)	Elastic–brittle	Good cytocompatibility; continuous cell layers line pore walls, though 3D colonization less pronounced than gyroid.

The PCL-DEW scaffold demonstrated the highest early proliferation (OD570 ∼2.5), with confocal imaging showing well-spread, elongated cells following aligned nanofibers. This superior performance is attributed to its ECM-like nanoscale architecture, which provides strong integrin engagement and enhanced mechanosensing. In comparison, the 3D-printed PCL scaffolds supported moderate growth (OD570 ∼1.7–1.8) and exhibited limited spreading along macrosized struts, reflecting the reduced availability of nanoscale anchoring features.

The Ossiform β-TCP scaffolds showed robust adhesion and favorable proliferation, particularly the gyroid (GY) and grid (GR) variants (OD570 ∼2.1 and ∼1.9, respectively). Their bioactive ceramic chemistry and microporosity facilitate ion exchange (Ca^2+^/PO_4_^3–^), promoting osteoconductive interactions and improved cellular engagement. Morphologically, cells adapted uniquely to each material: membrane-like on β-TCP surfaces, fiber-like on 3D-printed PCL, and sheath-like along PCL-DEW nanofibers.

In early osteogenic events, both stiffness and surface chemistry contribute, but in different ways. The β-TCP scaffolds benefit primarily from their surface chemistry, where Ca^2+^ and PO_4_^3–^ ions enhance integrin binding and early osteogenic signaling, whereas PCL scaffolds depend more on mechanical stiffness and nanoscale topography to generate cytoskeletal tension. The strong early response on PCL-DEW, therefore, reflects topography-driven mechanotransduction, while Ossiform β-TCP scaffolds rely on bioactive chemistry to stimulate early osteogenesis.

Overall, PCL-DEW performed best in early proliferation, Ossiform β-TCP provided the most biologically favorable interface for osteoconductive signaling, and 3D-printed PCL offered macroarchitectural precision with lower nanoscale guidance.

Although this study concludes at Day 7, longer-term cellular responses will likely diverge further as β-TCP undergoes ionic dissolution—enhancing osteogenic differentiation—and as PCL scaffolds gradually soften through hydrolytic degradation, altering mechanotransduction cues. The release of Ca^2+^/PO_4_^3–^ from β-TCP is expected to stimulate later-stage osteogenesis, while PCL degradation primarily modifies mechanical stiffness rather than chemistry. Extending studies to ≥28 days, including ALP activity and Alizarin Red staining, would therefore quantify how these dynamic changes regulate maturation and long-term mineralization.

While SEM and confocal imaging indicate greater cell spreading and deeper colonization—most notably on gyroid designs—these findings are primarily qualitative and may be affected by field-of-view selection and preparation artifacts (e.g., shrinkage during drying or surface masking that can resemble ECM deposition). Nevertheless, the combined morphology–viability trends provide a consistent, practical screening framework, helping to prioritize scaffold architectures (e.g., gyroid and PCL-DEW) for follow-up studies and streamlining the selection of the most promising designs for more detailed osteogenic evaluation.

## 4 Conclusions

This study systematically compared seven scaffold systems—PCL-DEW nanofibrous membranes, 3D-printed PCL architectures (grid, honeycomb, gyroid), and Ossiform β-TCP bioceramics—to elucidate how material composition and multiscale geometry jointly shape mechanical behavior and early osteogenic cell response. Across all platforms, our findings demonstrate that cellular outcomes are governed not by stiffness alone but by the interplay of nanoscale topography, architectural curvature, surface chemistry, and strain distribution, each of which contributes uniquely to early mechanotransduction. Among the tested systems, PCL-DEW consistently promoted the highest proliferation and most pronounced cell elongation, driven not by stiffness—which was the lowest among all scaffolds—but by its aligned nanoscale fibers that amplify integrin engagement, focal adhesion maturation, and cytoskeletal tension. Within the printed PCL lattices, the gyroid architecture enabled improved spreading and cell organization compared to grid and honeycomb designs, owing to its continuous curvature and strain-homogenizing geometry. Meanwhile, Ossiform β-TCP scaffolds supported robust adhesion and proliferation due to their bioactive ceramic surface, Ca^2+^/PO_4_^3–^ ion exchange, and microroughness, confirming that ceramic chemistry strongly influences early osteoconductive signaling. Although β-TCP is stiff and brittle at the macroscale, its surface chemistry and porosity promote early osteogenesis rather than inhibit it.

Mechanical testing revealed clear structure–mechanics relationships: grid PCL offered the highest stiffness, honeycomb provided balanced load-sharing, gyroid distributed strain more uniformly, and PCL-DEW was highly compliant yet biologically potent.

Overall, this study shows that optimal scaffold performance is achieved not by maximizing a single parameter, but by balancing mechanical competence with biological instruction. PCL-DEW is ideal for nanoscale guidance, β-TCP for osteoconductive chemistry, PCL-grid for structural support, and PCL-gyroid for curvature-driven cell organization. These complementary strengths highlight the promise of multimaterial, multiscale hybrid strategies, such as electrospin-spraying PCL nanofibers onto β-TCP gyroid scaffolds, to integrate flexibility, bioactivity, and mechanical stability within a single construct.

This study focused on early (day 7) responses, and future work should examine long-term osteogenic differentiation, mineralization, and the evolving mechanical environment as PCL softens under degradation and β-TCP undergoes ionic dissolution. Future studies will extend culture to ≥28 days, including ALP activity and Alizarin Red staining, to quantify osteogenic differentiation and matrix mineralization across these architectures. Testing in hydrated physiological media and under cyclic loading will further clarify scaffold behavior. Additionally, incorporating Live/Dead staining and longer-term differentiation markers as additional readouts would strengthen the biological assessment. Expanding beyond the current architectures to include additional fabrication methods—such as melt electrospin writing, graded porosity constructs, and composite PCL–ceramic hybrids—will be essential for capturing the hierarchical complexity of bone. Together, these directions underscore the necessity of exploring multiple scaffolding approaches to engineer next-generation biomimetic systems.

By integrating nano- to macroscale design principles across polymers and bioceramics, this work demonstrates that scaffold geometry and chemistry collectively dictate cell behavior, with gyroid architectures showing particularly favorable conditions for cell spreading and 3D colonization. The insights gained here provide a framework for designing multiscale, multimaterial scaffolds tailored to promote osteogenic performance and advance bone tissue engineering.

## Supplementary Material











## Notes

Figure S1: G-code and resultant grid structure mapped for direct electrospin writing; Figure S2: CAD renderings of the 3D-printed scaffold infill geometries—Grid, Gyroid, and Honeycomb; Figure S3: Load–displacement curve generated during mechanical testing of direct electrospunwritten PCL scaffolds; Figure S4: Tensile testing results showing elastic and viscoelastic behavior for PCL Grid, Gyroid, and Honeycomb patterned scaffold configurations; Figure S5: Load–displacement curve showing abrupt failure of the Ossiform scaffold after linear elastic deformation; Figure S6: Indirect cytotoxicity of scaffold-conditioned media on L929 cells (MTT, 24 h % cell survival; Figure S7: Direct electrospun written PCL-(DEW) scaffolds for direct culture tests with Mg63 osteosarcoma cells; Figure S8: Direct culture cell tests of Mg63 human osteosarcoma cells on 3D-printed PCL scaffolds; Figure S9: Results of direct cell culture tests of Mg63 human osteosarcoma cells on Ossiform β-TCP scaffolds; Figure S10: Day 7 Confocal Imaging of cell-seeded PCL-DEW scaffold; Figure S11: Day 7 Confocal imaging of cell-seeded Ossiform GY β-TCP scaffold (PDF)
Cell culture tests (XLSX)
Raw mechanical data sets (XLSX)
Printing process (MP4)
Printing process (MP4)
