# Supplementary material for: Scaffold Material–Architecture Design Rules Linking Mechanics and Early Osteogenesis in PCL/β-TCP Grid, Honeycomb, and Gyroid Lattices
Source: ACS Omega. 2026 Apr 22;11(17):25088–100. doi: 10.1021/acsomega.5c10393 (PMC13150591; doi:10.1021/acsomega.5c10393)
Supplement: Supplementary file 1 [file ao5c10393_si_001.pdf]

Scaffold Material–Architecture Design Rules Linking Mechanics and Early Osteogenesis in PCL/β-TCP Grid, Honeycomb, and Gyroid Lattices

Shweta Thapa*, Pete Twigg^, Maria Katsikogianni[±], Dimitra Tsaroucha[β], Mitali Singhal[∞]

*** Medical & Healthcare Technology, Faculty of Engineering & Digital Technologies, University of Bradford, England, UK BD7 1DP**

^Faculty of Engineering and Digital Technologies, University of Bradford, England, UK BD7 1DP

[±]School of Chemistry, Faculty of Life Sciences, University of Bradford, England, UK BD7 1DP

[β]Biomedical Science, Faculty of Life Science, University of Bradford, England, UK BD7 1DP

[∞]Institute of Cancer Therapeutics, University of Bradford, England, UK BD7 1DP

Supporting Information

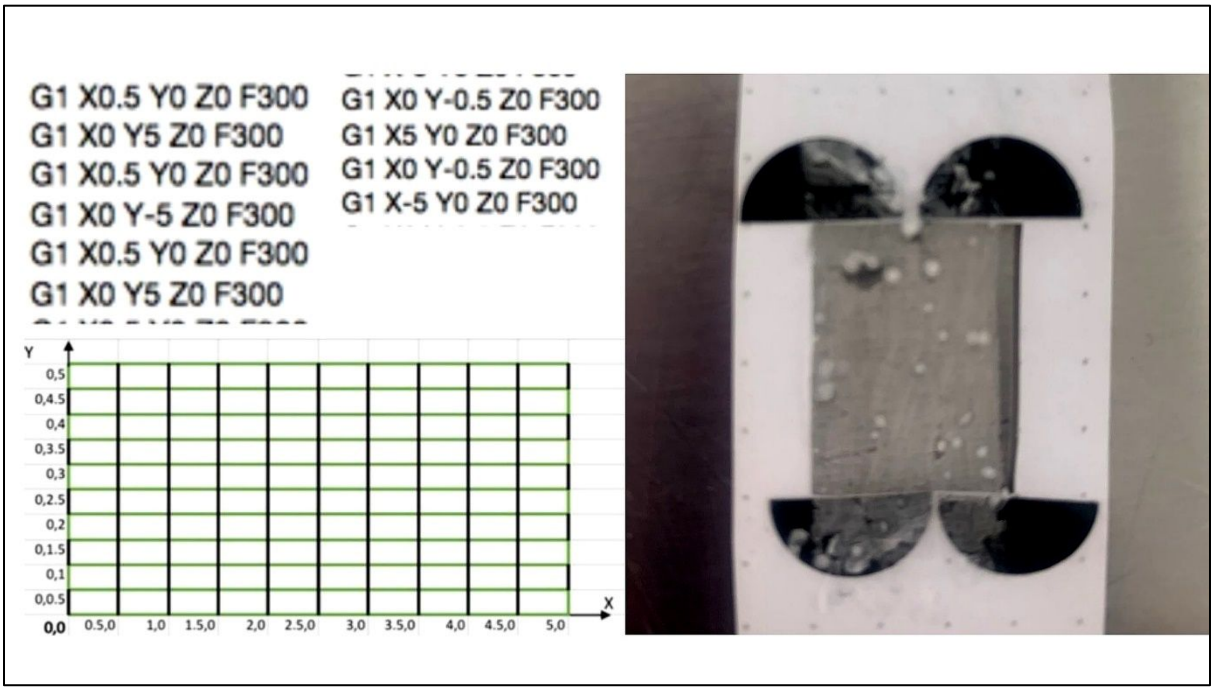

Figure S1: G-code and resultant grid structure mapped for direct electrospin writing with 500 µm X-Y spacing (Left), PCL DEW scaffold printed (Right).

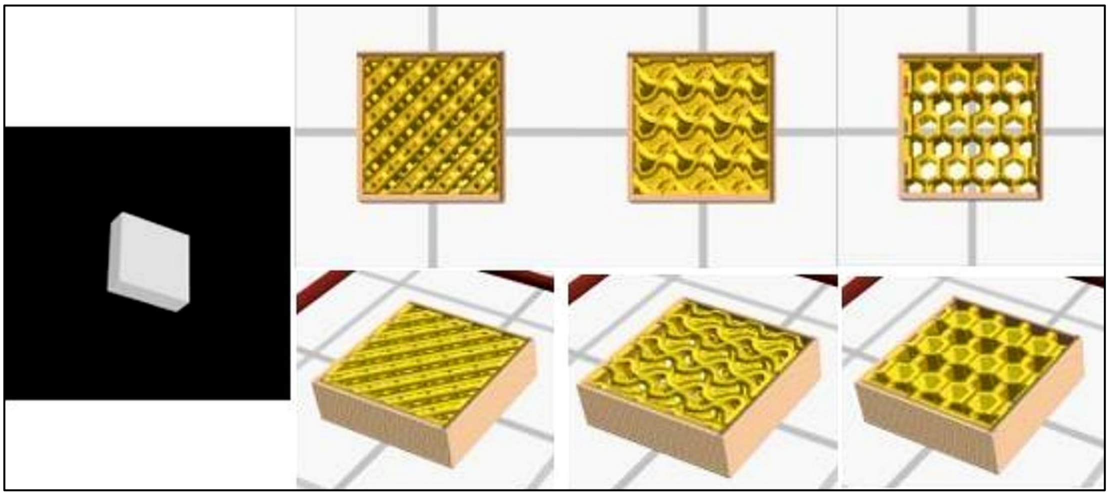

Figure S2: CAD renderings of the 3d printed scaffold infill geometries—Grid, Gyroid, and Honeycomb (Left to Right).

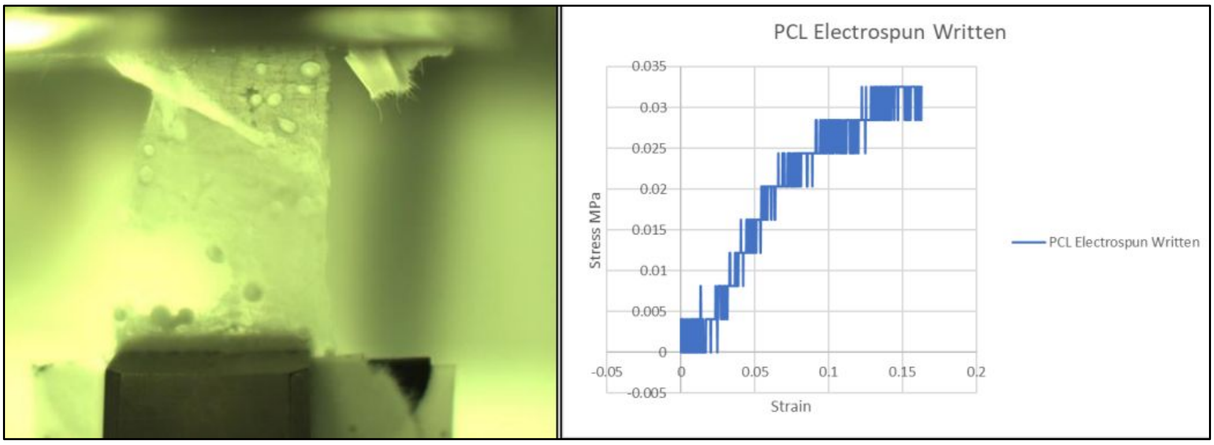

Figure S3: Load-displacement curve generated during mechanical testing of direct electrospun-written PCL scaffolds.

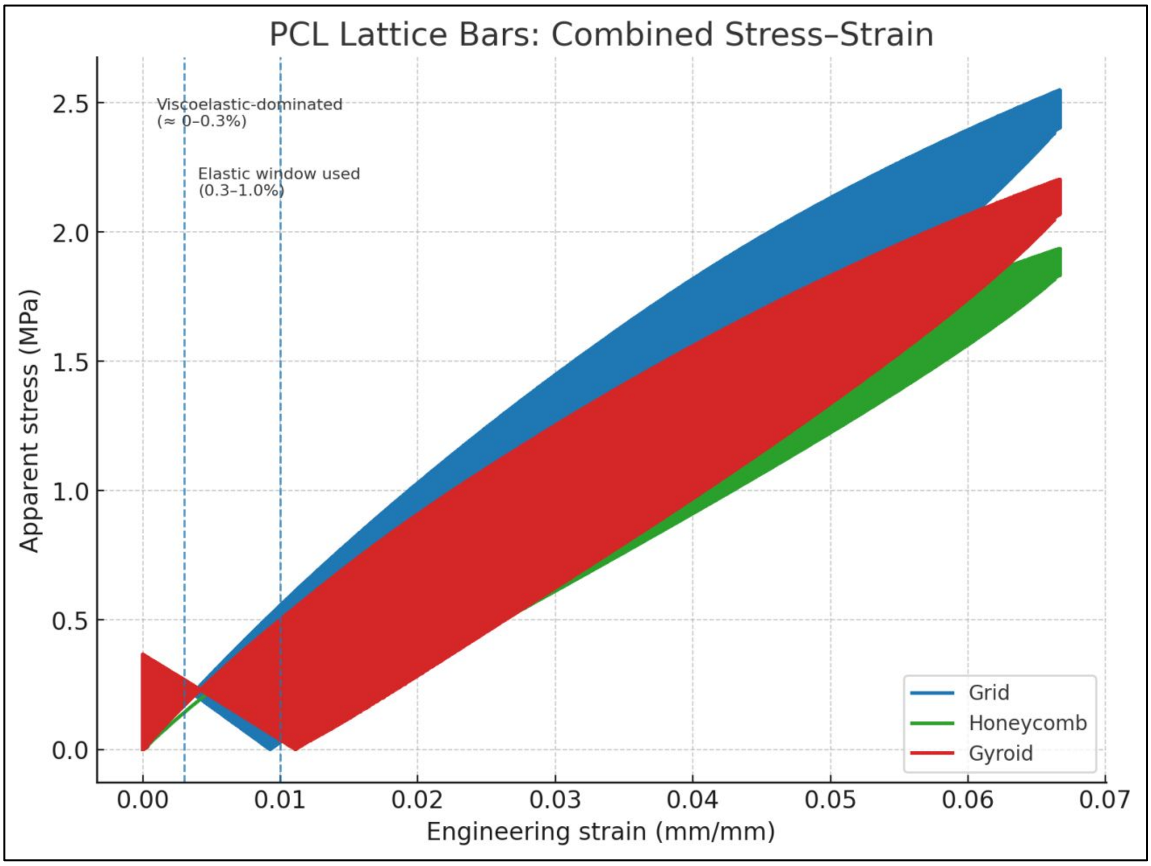

Figure S4: Tensile testing results showing elastic and viscoelastic behaviour for PCL Grid, Gyroid, and Honeycomb patterned scaffold configurations.

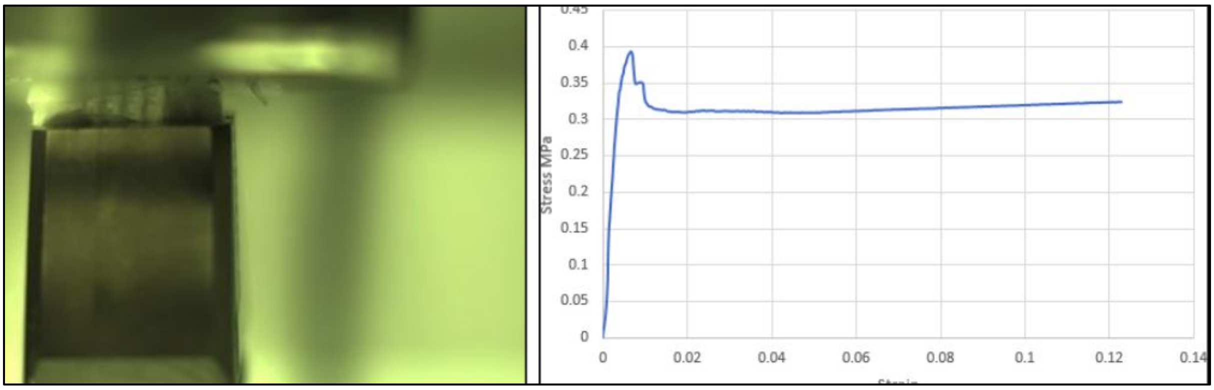

Figure S5: Load–displacement curve showing abrupt failure of the Ossiform scaffold after linear elastic deformation.

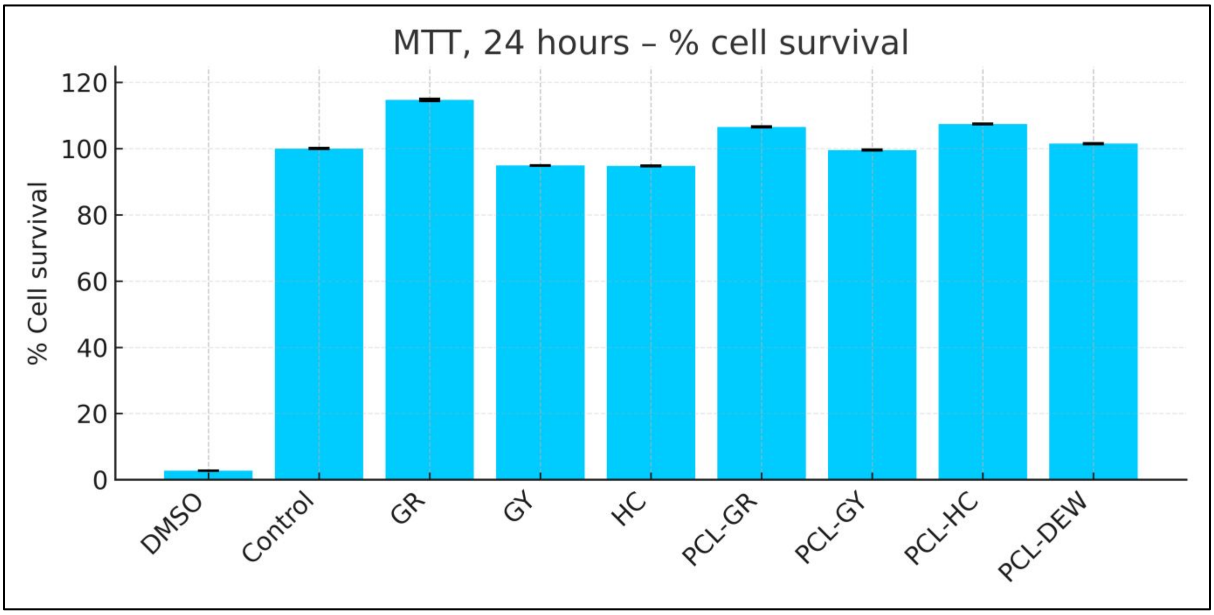

Figure S6: Indirect cytotoxicity of scaffold-conditioned media on L929 cells (MTT, 24 h % cell survival

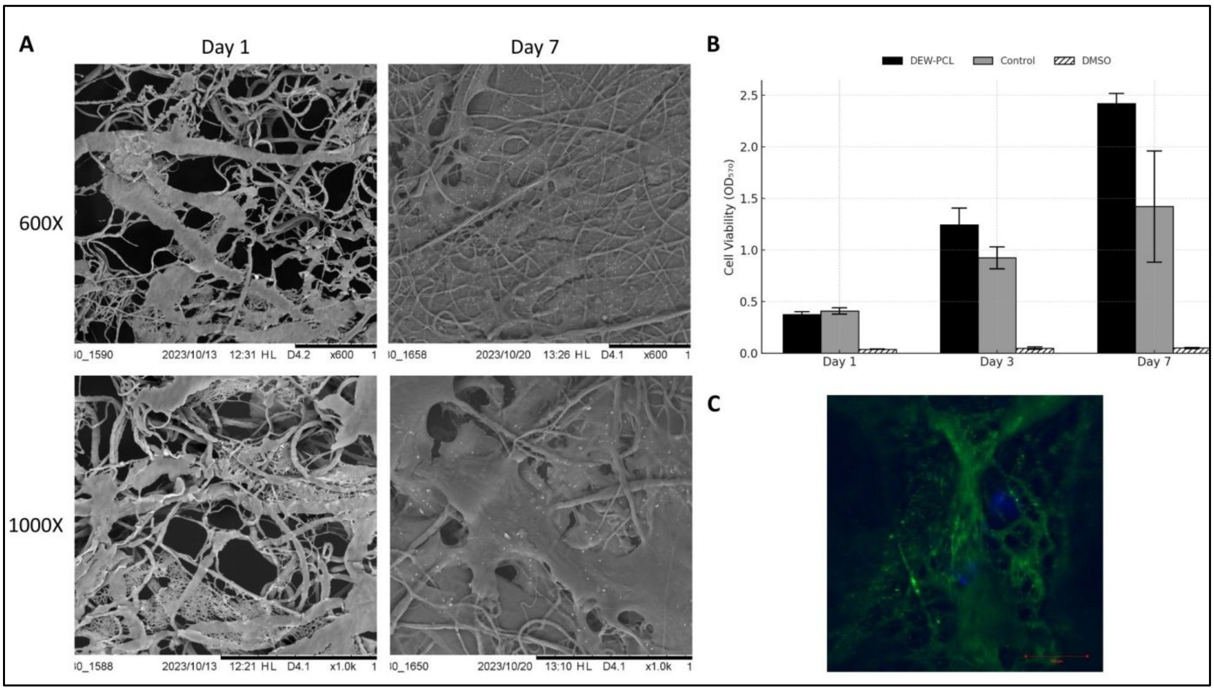

Figure S7: Direct Electrospun Written PCL-(DEW)Scaffolds for Direct culture tests with Mg63 Osteosarcoma cells(A) Progression of osteogenic activity on DEW nanoscaffolds from day 1 to day 7 (B) MTT Assay (Cell viability) results for DEW-PCL on day 1,3 and 7 compared against Control & DMSO (C) Immunofluorescent(Confocal) image of DEW scaffold day7 showing actin filaments elongating(green, phalloidin) and nuclei spots(DAPI)

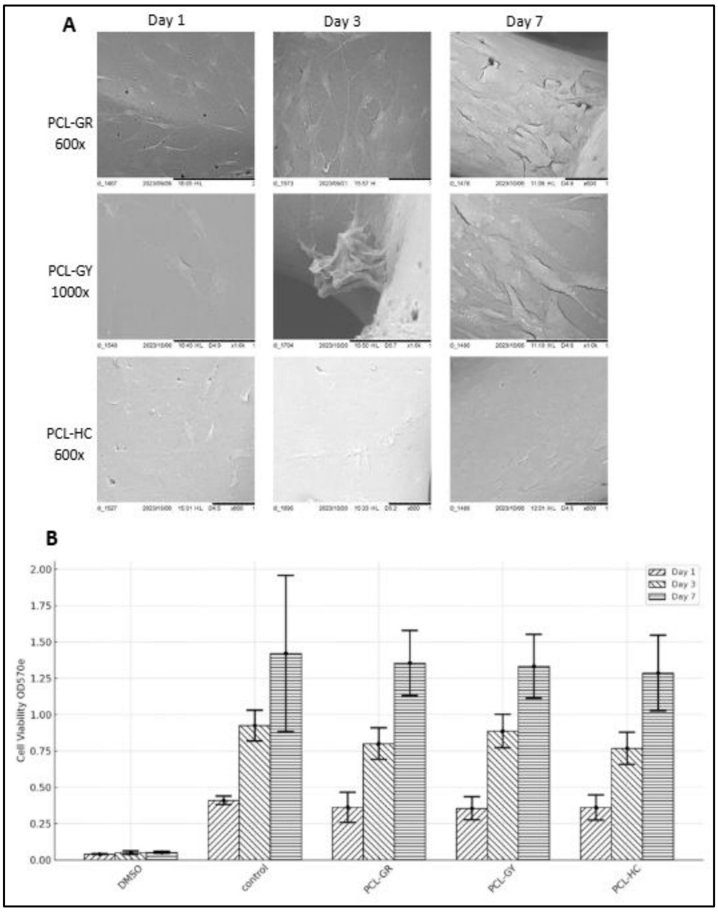

Figure S8: scaffold (f direct culture cell tests of Mg63 human osteosarcoma cells on 3D printed PCL scaffolds (A) Day 1,3 and 7 progression for Grid scaffold(PCL-GR),Gyroid scaffold(PCL-GY), Honeycomb scaffold (PCL-HC)(B) Cell Viability MTT tests for Grid scaffold(PCL-GR),Gyroid scaffold(PCL-GY), Honeycomb scaffold (PCL-HC) compared against Control and DMSO

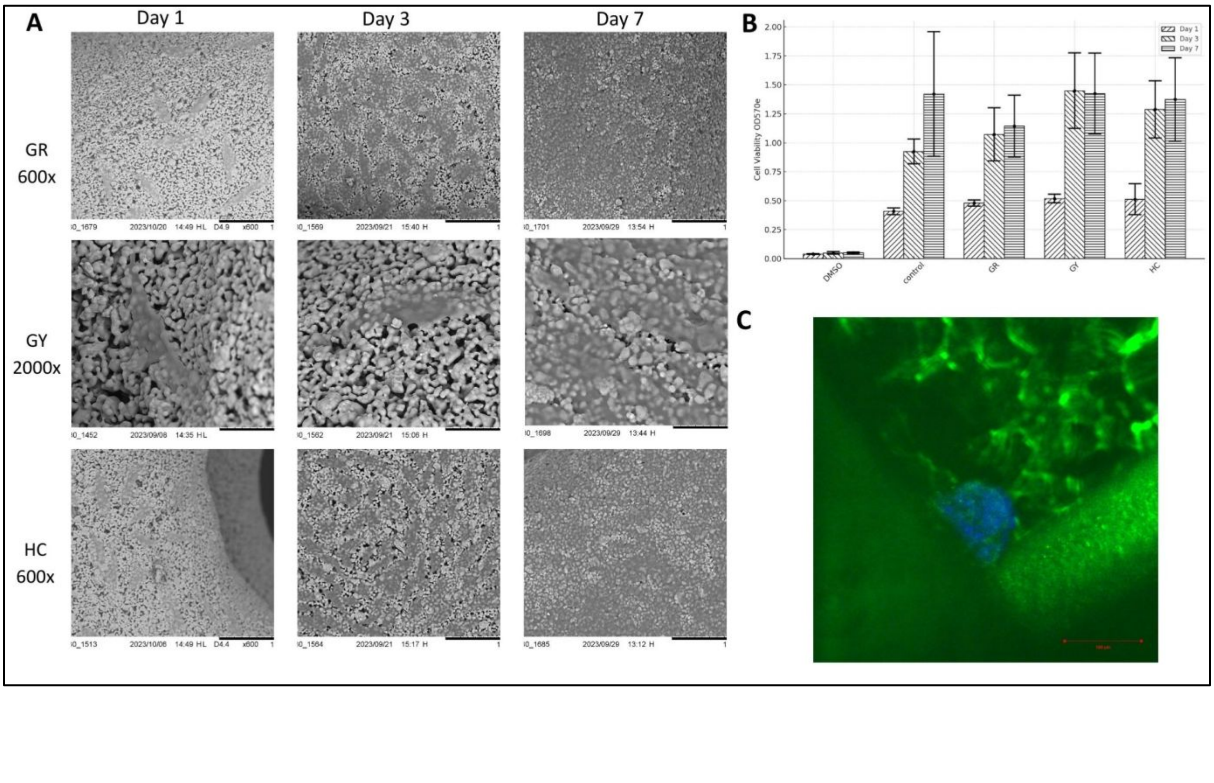

Figure S9: Results of direct cell culture tests of Mg63 human osteosarcoma cells on Ossiform β-TCP scaffolds (A) Day 1,3 and 7 progression for Ossiform Grid scaffold(GR),Ossiform Gyroid scaffold(GY), Ossiform Honeycomb scaffold (HC)(B) Cell Viability MTT tests for Ossiform Grid scaffold(GR),Ossiform Gyroid scaffold(GY), Ossiform Honeycomb scaffold (HC)compared against Control and DMSO (C) Immunofluorescent Ossiform Grid scaffold (GR) showing nuclei (DAPI staining) along the struts and actin filaments(phalloidin staining)showing cytoskeletal organization, adhesion & potential infiltration into pores, encouraging 3D colonization

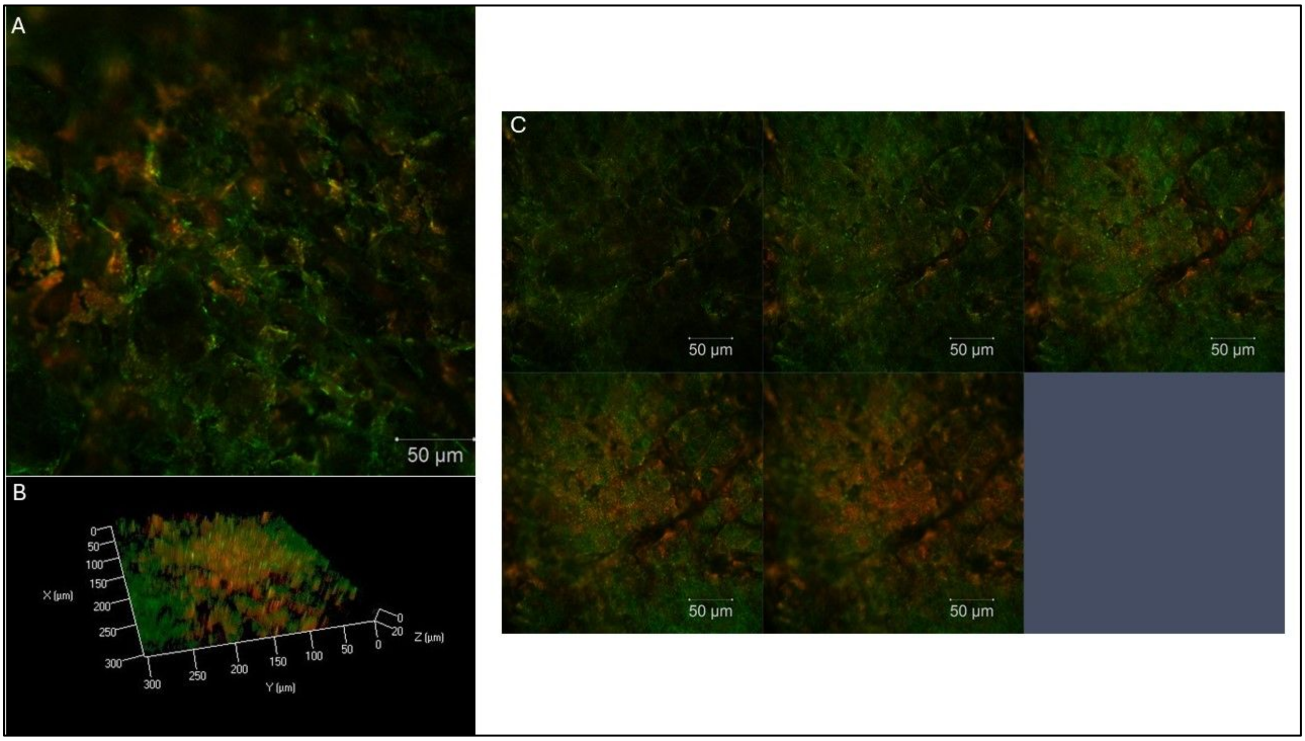

Figure S10: Day 7 Confocal Imaging of cell-seeded PCL-DEW scaffold (A) dense cell coverage, viability, and penetration across the scaffold surface (B) 3D reconstructed z stack of PCL-DEW scaffold (C) orthogonal optical slices demonstrating cell distribution through the scaffold depth.

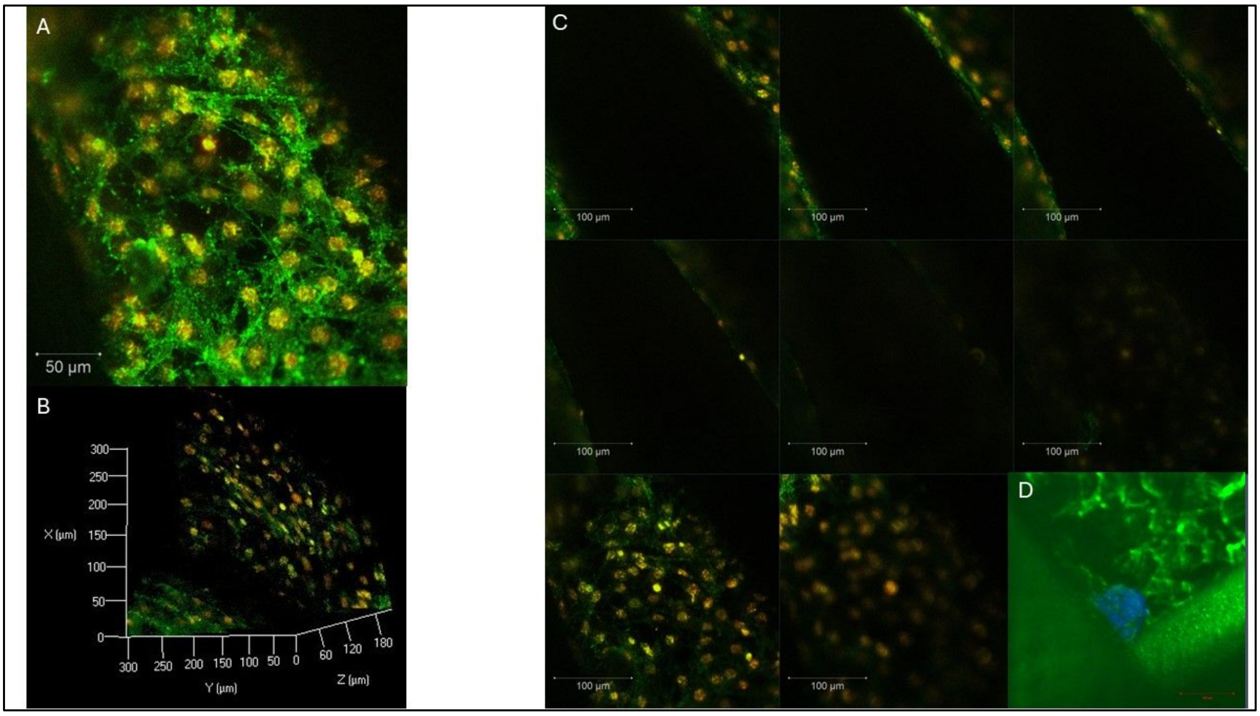

Figure S11: Day 7 Confocal imaging of cell-seeded Ossiform GY β-TCP scaffold. A) Maximum-intensity projection of actin (green) and nuclei (yellow/orange) showing dense cellular colonization and spreading across the gyroid pore walls. (B) 3D z-stack reconstruction illustrating multilayer cell infiltration throughout the gyroid architecture and curvature-guided cytoskeletal organization. (C) Representative optical slices from the z-stack (orthogonal views) confirming the presence of cells at multiple depth planes within the scaffold pores. The layered nuclear distribution demonstrates 3D mechanosensing and pore infiltration. (D) High-magnification single-plane image highlighting a cell nucleus (DAPI, blue) located within a recessed gyroid pore. The spherical DAPI appearance results from optical sectioning through a curved pore cavity rather than an imaging artifact, consistent with the 3D geometry of β-TCP gyroid scaffolds.
